# Strengthening healthcare systems to prevent antimicrobial resistance in LMICs: it is time to act

**DOI:** 10.7189/jogh.16.03007

**Published:** 2026-03-13

**Authors:** Virak Sorn

**Affiliations:** Faculty of Health Sciences and Biotechnology, University of Puthisastra, Phnom Penh, Cambodia

## Abstract

Antimicrobial resistance (AMR) poses a rapidly growing threat to global health, with low- and middle-income countries (LMICs) bearing a disproportionate burden. These LMICs face challenges such as weak health system governance, limited diagnostic capacity, undertrained workforces, and poorly regulated antibiotic markets that drive inappropriate antimicrobial use and accelerate AMR. In settings where over-the-counter antibiotic sales remain widespread and empirical treatment is a routine, AMR undermines treatment effectiveness and increases preventable morbidity and mortality. If left unaddressed, AMR is predicted to result in 10 million deaths per year by 2050. Combating AMR in LMICs requires a comprehensive approach: strengthening healthcare systems, enforcing prescription-only policies, raising public health awareness, and fostering collaboration between governments, non-governmental organisations, and international stakeholders. Priority actions include regulating informal pharmaceutical markets, embedding antimicrobial stewardship within primary care, expanding access to basic diagnostics, and using digital health tools to support prescribers and patients. Drawing on regional LMIC experiences, this viewpoint highlights practical pathways for aligning health system reform with AMR containment.

Antimicrobial resistance (AMR) is widely recognised as one of the most serious global public health threats of the 21st century, with low- and middle-income countries (LMICs) experiencing a disproportionate share of resistant infections and AMR-related mortality [1,2]. Although antibiotics have revolutionised the management of infectious diseases, their inappropriate and excessive use – particularly in settings with weak regulatory and health system oversight – has accelerated resistance and undermined treatment effectiveness worldwide [1–4].

Antibiotics are readily available without prescription in many LMICs through private pharmacies, informal drug vendors, and poorly regulated markets [3–5]. The circulation of substandard and falsified medicines, widespread antibiotic use in agriculture, inadequate surveillance systems, malnutrition, recurrent infections, and limited access to quality healthcare collectively exacerbate the AMR burden in these settings [4,5]. Simultaneously, limited diagnostic capacity and shortages of trained healthcare personnel encourage empirical prescribing practices, often involving broad-spectrum antibiotics as first-line therapy [6]. These structural constraints, rather than prescribed behaviour alone, form the foundation of AMR in resource-constrained environments.

Improved access to antibiotics in LMICs has undoubtedly enhanced treatment outcomes for infectious diseases. However, inappropriate use – including self-medication, incomplete treatment courses, and poor adherence to medical advice – has contributed to the rapid growth of resistance [5]. Such practices are especially common among populations with limited health literacy and inadequate hygiene conditions in rural and underprivileged urban communities. These behaviours are closely linked to systemic gaps in regulation, education, and healthcare delivery, rather than individual negligence.

The global magnitude of AMR underscores its urgency; as reported by the Centers for Disease Control and Prevention in 2022 [5], antibiotic resistance was linked to around 5 million deaths and directly killed at least 1.27 million people globally in 2019, making it a major public health concern worldwide [5,7]. Projections indicate that, if current trends persist, LMICs may account for up to 80% of the anticipated 10 million annual AMR-related deaths by 2050 [8]. Frontline healthcare providers in these settings must manage infectious diseases within underfunded systems with limited infrastructure, inadequate diagnostics, and insufficient professional training [4,5,9]. As a result, empirical treatment becomes a practical necessity, rather than a clinical preference, inadvertently driving the emergence of drug-resistant microorganisms.

Further compounding these challenges is the limited awareness and implementation of antimicrobial stewardship frameworks such as the World Health Organization’s AWaRe classification [10]. In many LMICs, stewardship is hindered by the absence of context-specific guidelines, monitoring systems, and institutional accountability. Patients colonised with multidrug-resistant organisms – including methicillin-resistant *Staphylococcus aureus*, vancomycin-resistant *Enterococcus*, *Clostridioides difficile*, drug-resistant tuberculosis, and malaria – face significantly higher risks of treatment failure and mortality in both community and healthcare settings. These clinical risks are amplified by socioeconomic vulnerabilities that facilitate transmission and limit access to effective treatment [9–11].

Health emergencies further expose the fragility of healthcare systems in LMICs. Outbreaks of communicable diseases disrupt supply chains, overwhelm limited diagnostic capacity, and divert attention from antimicrobial stewardship efforts [12]. Under such pressures, health care providers must navigate competing priorities while attempting to maintain appropriate antibiotic use.

## HEALTH SYSTEM DRIVERS OF AMR IN LMICS

AMR in LMICs is not merely a consequence of individual antibiotic misuse; it is driven by system-level weaknesses across health services, governance, regulation, and socioeconomic structures. Evidence indicates that there were several interconnected system failures that contributed to creating an enabling environment for resistance selection, transmission, and escalation [3–6,10,13–24].

### Weak regulation and over-the-counter antibiotic access

One of the most consistent health system drivers of AMR in LMICs is the poor regulation of antibiotic distribution and use. In many LMICs, antibiotics are widely available without prescription from pharmacies, informal vendors, and unregulated markets [3–5]. This unrestricted access facilitates overuse, self-medication, and inappropriate dosing, all of which increase selective pressure for resistant organisms. Studies have shown that regulatory enforcement of prescription-only policies is patchy or absent, and in some LMIC cities, up to 60% of antibiotic sales occur without professional medical consultation [13–15]. Additionally, self-medication practices are highly prevalent and driven by structural impediments to formal care, including cost, geographical barriers, and poor healthcare access [6,15,16].

Conversely, over-the-counter access and poor-quality drugs contribute directly to misuse and resistance selection. Informal healthcare providers and drug sellers constitute a significant part of care delivery in LMIC communities. Their practices – including dispensing without prescription and symptomatic treatment with antibiotics – drive misuse in the absence of regulatory oversight and structured training [17].

### Limited diagnostic capacity and laboratory infrastructure

Diagnostic deficiencies within health systems represent a fundamental driver of AMR in LMICs. Many healthcare facilities lack reliable laboratory services and rapid diagnostic tools needed to differentiate bacterial from viral infections and identify causative pathogens and their susceptibility patterns. In the absence of such capacities, clinicians are compelled to rely on empirical treatment, frequently defaulting to broad-spectrum antibiotics [18]. Multiple reports across LMICs consistently highlight that limited diagnostic capability, combined with constrained access to appropriate antimicrobials, leads to inappropriate antibiotic use and suboptimal treatment decisions [18–20]. Strengthening diagnostic services is, therefore, a critical structural requirement for rational antimicrobial use; however, many facilities continue to lack basic microbiology laboratories and antimicrobial susceptibility testing capabilities.

### Poor implementation of antimicrobial stewardship

Antimicrobial stewardship programmes (ASPs), central to optimising antibiotic use, remain inadequately implemented across many health systems in LMICs. Limited adoption of stewardship frameworks, including the WHO AWaRe classification, and the absence of context-specific guidelines, monitoring mechanisms, and accountability structures undermine rational prescribing practices [10]. Stewardship is frequently misperceived as a tertiary-care responsibility, rather than a core component of primary healthcare. Evidence from global surveys further indicates that national stewardship efforts are inconsistent, with many LMICs lacking coordinated ASP strategies in human medicine [21].

Several hospital-based research studies further identify barriers to effective antimicrobial stewardship, including limited leadership engagement, inadequate training of health care workers, and low institutional prioritisation of ASP, even in settings where programmes are formally established [22–24]. Moreover, although prescription-only regulations exist in policy, weak enforcement in practice reveals persistent gaps between legislation and implementation, contributing to irrational antibiotic use. These challenges are often intensified during health emergencies, when system pressures further compromise prescribing oversight.

## MOVING FROM GENERIC HEALTH SYSTEM STRENGTHENING TO TARGETED ACTION

Addressing AMR in LMICs requires more than broad appeals to strengthen health systems; it necessitates precise, enforceable reforms that emphasise antimicrobial stewardship, effective pharmaceutical regulation, and support for frontline clinical decision-making. Although the call to ‘strengthen health systems’ is frequently invoked in AMR discussions, it often lacks practical and operational clarity. Effective AMR control in LMICs should, therefore, concentrate on three actionable priority areas: enforcing regulation of antibiotic markets, embedding antimicrobial stewardship within primary care, and leveraging digital health technologies to support decision-making.

### Enforcing regulation of antibiotic markets

Regulatory enforcement is essential to control inappropriate antibiotic access, which drives misuse and resistance. Evidence from LMICs shows that despite existing policies, non-prescription antibiotic sales remain widespread [6,8,11–15,25,26]. A scoping review using the WHO AWaRe classification highlighted that many LMICs, including Bangladesh, Pakistan, and Ghana, struggle to curtail over-the-counter antibiotic sales, with simulated client studies reporting antibiotics dispensed without a prescription in up to 70% of visits [25]. While another study in Cambodia also illustrated that due to communities wanting easy access to antibiotics, there remains a demand for services provided by invisible and informal health care practices that have inadequate knowledge of antibiotic use [26]. Such findings underscore persistent gaps between regulatory frameworks and their enforcement, contributing to inappropriate use outside formal clinical settings. Strengthening regulation, therefore, requires not only prescription-only policies, but also inspection mechanisms, enforcement systems, penalties for non-compliance, and engagement of informal drug sellers through licensing and training so they can be integrated into regulated supply chains, reducing unsafe practices that currently undermine regulatory efforts.

### Embedding antimicrobial stewardship within primary care

Primary care represents the first point of contact for most patients and the setting where most antibiotic prescriptions originate. Although ASPs aim to optimise antibiotic use and reduce misuse, their implementation in LMICs remains limited, particularly at the primary care level. Evidence indicates that ASP interventions – ranging from clinical training to structured guidelines – can significantly improve prescribing practices in LMICs and achieve success when bundled with education, diagnostic support, and guideline enforcement, although resource limitations remain a major barrier [22,27]. However, most ASP efforts to date have been concentrated in hospital settings, and primary care stewardship remains underdeveloped, reflecting a missed opportunity for early intervention where most antibiotics are prescribed. These findings also indicate barriers to stewardship implementation in hospitals, such as inadequate leadership support, limited training, insufficient monitoring, and a lack of national guidelines, that similarly hinder uptake at the primary care level [22,27–29]. To overcome these challenges, stewardship must be embedded into primary healthcare through simplified treatment guidelines, task-specific training for frontline providers, and audit-and-feedback systems that reinforce rational prescribing practices [27–29].

### Leveraging digital health technologies

Digital health technologies have emerged as promising tools to support antibiotic stewardship and improve prescribing quality, offering scalable solutions even in resource-limited settings. Use of digital health technologies, including telepharmacy, mobile health (mHealth) applications, electronic decision-support systems and other digital tools, to reinforce guideline-concordant care and provide real-time clinical support and to support patient education [30–35]. As previous reports have highlighted, digital tools such as computerised decision support and mHealth applications can promote adherence to antibiotic guidelines and facilitate stewardship efforts, particularly when integrated with monitoring and feedback mechanisms [31–33]. For example, electronic decision-support systems can provide clinicians with real-time information on appropriate antibiotic selection, dose optimisation, and de-escalation, while telepharmacy services can extend stewardship principles to remote or underserved populations. Adapting these tools for LMICs requires accounting for infrastructure limitations and gaps in digital literacy; without careful design, digital innovations risk widening inequities instead of alleviating them. Nevertheless, when digital solutions are tailored to local capacity and integrated into routine clinical workflows, they can extend stewardship principles across diverse health care settings and to improve antibiotic prescribing practices sustainably.

## LIMITATIONS

This viewpoint relies primarily on secondary literature and regional examples, rather than original empirical data. Given the heterogeneity of LMICs, the feasibility of proposed interventions will vary according to governance capacity, financing, and health system maturity. Nonetheless, the structural drivers identified are consistently reported across diverse LMIC contexts and provide a practical framework for policy prioritisation.

## CONCLUSIONS

AMR in LMICs is not solely a clinical problem, but is rather a manifestation of deeper systemic weaknesses in healthcare regulation, infrastructure, and clinical decision support. Effective responses, therefore, require moving beyond broad rhetoric on health system strengthening toward targeted, enforceable reforms that prioritise pharmaceutical regulation, antimicrobial stewardship, and support for frontline prescribing practices. Without decisive measures – particularly to control over-the-counter antibiotic sales and to integrate stewardship into primary care – AMR will continue to undermine the achievements of modern medicine and disproportionately affect vulnerable populations.
